# Interacting Multiple Model (IMM) Fifth-Degree Spherical Simplex-Radial Cubature Kalman Filter for Maneuvering Target Tracking

**DOI:** 10.3390/s17061374

**Published:** 2017-06-13

**Authors:** Hua Liu, Wen Wu

**Affiliations:** Ministerial Key Laboratory of JGMT, Nanjing University of Science and Technology, Nanjing 210094, China; wuwen@njust.edu.cn

**Keywords:** maneuvering target tracking, interacting multiple model, fifth-degree spherical simplex-radial rule, Markov process

## Abstract

For improving the tracking accuracy and model switching speed of maneuvering target tracking in nonlinear systems, a new algorithm named the interacting multiple model fifth-degree spherical simplex-radial cubature Kalman filter (IMM5thSSRCKF) is proposed in this paper. The new algorithm is a combination of the interacting multiple model (IMM) filter and the fifth-degree spherical simplex-radial cubature Kalman filter (5thSSRCKF). The proposed algorithm makes use of Markov process to describe the switching probability among the models, and uses 5thSSRCKF to deal with the state estimation of each model. The 5thSSRCKF is an improved filter algorithm, which utilizes the fifth-degree spherical simplex-radial rule to improve the filtering accuracy. Finally, the tracking performance of the IMM5thSSRCKF is evaluated by simulation in a typical maneuvering target tracking scenario. Simulation results show that the proposed algorithm has better tracking performance and quicker model switching speed when disposing maneuver models compared with the interacting multiple model unscented Kalman filter (IMMUKF), the interacting multiple model cubature Kalman filter (IMMCKF) and the interacting multiple model fifth-degree cubature Kalman filter (IMM5thCKF).

## 1. Introduction

Maneuvering target tracking has been widely used in many applications, such as aircraft surveillance [1,2], road vehicle navigation [3,4] and radar tracking [5,6,7]. Because of the complexity of maneuvering target motion, the single model structure is not appropriate for tracking maneuvering targets. Therefore, the multiple-model structure is adopted. A number of multiple-model techniques have been proposed, such as multiple model (MM) methods [8], optimization of the multiple model neural filter [9], the interacting multiple model (IMM) algorithm [10,11], and other algorithms [12,13]. In these multiple model algorithms, the IMM algorithm proposed by Blom and Bar-Shalom [10,11] is the most popular algorithm. In the IMM algorithm, the target model is selected among a set of models via the control of a Markov chain and the final estimate is obtained by a weighted sum of the estimates from the sub-filters of different models. The conventional IMM algorithm combines multiple models with a linear filter to estimate the target motion state. Because of its excellent compromise between complexity and perfor, the IMM [8,14] algorithm has been widely used in the field of maneuvering target tracking [14,15,16]. However, in the conventional IMM algorithm, the Kalman filter only obtains high precision for linear Gaussian systems. However, most modern systems are nonlinear and the linear IMM algorithm cannot directly deal with nonlinear systems. Thus, the research of nonlinear IMM is paid more attention and is a popular topic in maneuvering target tracking field. It is different from linear IMM theory based on the linear Kalman filter; the performance of nonlinear IMM algorithms depends on the selected nonlinear filters.

Nowadays, the study of nonlinear filter algorithms has been paid a great deal of attention by researchers. It is well known that the extended Kalman filter (EKF) [17] is widely used among the proposed nonlinear filtering algorithms. The basic idea of the EKF is to linearize the measurements and state models by first-order Taylor series expansion. However, it is difficult to get the Jacobian matrix of nonlinear function in many practical problems. As a result, the performance of the EKF may degrade rapidly. To solve this problem, scholars have proposed derivative-free alternatives such as the unscented Kalman filter (UKF) [18,19], the central difference Kalman filter (CDKF) [20] and the Gauss-Hermite Kalman filter (GHKF) [21], etc. These algorithms mentioned above use a set of deterministic sampling points and weights to approximate the Gaussian integrals, which are more accurate than the EKF. However, the search for more accurate filtering algorithms is continuing. In recent years, the cubature Kalman filter (CKF) [22,23] has been of increasing interest for high-dimensional state estimation. This filtering algorithm approximates the weighted Gaussian integrals according to the Bayesian theory and the third-degree spherical-radial cubature rule.

To further improve the estimation accuracy of CKF, the fifth-degree CKF (5thCKF) is proposed in [24]. However, the computational cost of the 5thCKF increases rapidly with the increasing of the state dimension. Recently, Wang et al. [25] have proposed a new class of CKF algorithms based on the spherical simplex-radial (SSR) rule, which can improve accuracy of the CKF with lower computational costs in high dimensional nonlinear system. Specially, the fifth-degree spherical simplex-radial cubature Kalman filter (5thSSRCKF) proposed in [25] has a higher estimation accuracy than 5thCKF. Therefore, we choose the 5thSSRCKF as the filtering algorithm in the IMM framework, and propose the interacting multiple model fifth-degree spherical simplex-radial cubature Kalman filter (IMM5thSSRCKF) algorithm for maneuvering target tracking of nonlinear system. Simulation results show that the IMM5thSSRCKF exhibits better performance than the interacting multiple model unscented Kalman filter (IMMUKF), the interacting multiple model cubature Kalman filter (IMMCKF) and the interacting multiple model fifth-degree cubature Kalman filter (IMM5thCKF) [26] in terms of accuracy and switching response.

The remainder of this paper is organized as follows. The fifth-degree spherical simplex-radial cubature Kalman filter is briefly reviewed in Section 2. The whole procession of IMM5thSSRCKF used in target tracking problem is developed in Section 3. Simulation results of a maneuvering tracking problem and performance comparisons are presented and discussed in Section 4. Finally, the conclusions are provided in Section 5.

## 2. Fifth-Degree Simplex-Spherical Cubature Kalman Filter

The nonlinear filtering problem with additive process and measurement noise can be defined as:

(1) $$\left\{ \begin{array}{l} x_{k}=f\left(x_{k-1}\right)+w_{k-1}\\ z_{k}=h\left(x_{k}\right)\;+v_{k} \end{array}\right.$$

where k is a discrete time index, $x_{k}\in R^{n}$ is the state vector at time k, $z_{k}\in R^{m}$ is the measurement vector at time k; f(⋅) and h(⋅) are the system dynamics function and the measurement function; $w_{k-1}\in R^{n}$ is the process noise; and $v_{k}\in R^{m}$ is the measurement noise. $w_{k-1}$ and $v_{k}$ are assumed to be uncorrelated zero-mean Gaussian white noise with covariance matrix $Q_{k-1}$ and $R_{k}$, respectively. The initial state $x_{0}$ is assumed to be ${\hat{x}}_{0}$ with covariance matrix $P_{0}$ and is independent of $w_{k-1}$ and $v_{k}$.

### 2.1. Review of the Fifth-Degree Spherical Simplex-Radial Cubature Rule

The 5thSSRCKF algorithm has the same structure as the general Gaussian approximation filters, such as the CKF, but uses the fifth-degree spherical simplex-radial cubature rule to calculate the Gaussian weight integral $I(f)=\int_{R^{n}}f(x)N(x;0,I)dx$. By using the spherical simplex-radial cubature rule, the 5thSSRCKF method can get more accurate estimation than CKF. In the fifth-degree spherical simplex-radial cubature rule, the following integral is considered [24]:

(2) $$I(f)=\int_{R^{n}}f(x)\exp(-x^{T}x)dx$$

where f(⋅) is arbitrary nonlinear function, and ${\mathbb{R}}^{n}$ is the integral domain. To calculate the above integral, let x=rs $\left(s^{T}s=1,r=\sqrt{x^{T}x}\right)$. Equation (2) can be transformed into the spherical-radial coordinate system:

(3) $$I(f)=\int_{0}^{\infty }\int_{U_{n}}f(rs)\;r^{n-1}\exp(-r^{2})d\sigma (s)\;dr$$

where $s={\left[s_{1},s_{2},\cdots ,s_{n}\right]}^{T}$, $U_{n}=\{s\in {\mathbb{R}}^{n}:s_{1}^{2}+s_{2}^{2}+\cdots +s_{n}^{2}=1\}$ is the *n*-dimensional spherical surface, and σ(⋅) is the area element on $U_{n}$; *n* denotes the dimension of spherical surface. Then, the integral (3) can be decomposed into the spherical integral $S(r)=\int_{U_{n}}f(rs)d\sigma (s)$ and the radial integral $I(f)=\int_{0}^{\infty }S(r)r^{n-1}\exp(-r^{2})dr$.

#### 2.1.1. Spherical Simplex Rule

As can be seen from [27], the spherical integral $\int_{U_{n}}f(rs)d\sigma (s)$ can be approximated by the transformation group of the regular *n*-simplex. The fifth-degree spherical simplex rule with $n^{2}+3n+2$ quadrature points is given by:

(4) $$S_{5}(r)=\frac{(7-n)n^{2}A_{n}}{2{\left(n+1\right)}^{2}{\left(n+2\right)}^{2}}\sum_{j=1}^{n+1}\left[g(ra_{j})+g(-ra_{j})\right]+\;\frac{2{\left(n-1\right)}^{2}A_{n}}{2{\left(n+1\right)}^{2}{\left(n+2\right)}^{2}}\sum_{j=1}^{n(n+1)/2}\left[g(rb_{j})+g(-rb_{j})\right]$$

where the surface area of the unit sphere is $A_{n}=2\sqrt{\pi^{n}}/\Gamma^{n}(1/2)$. The points sets of $a_{j}$ and $b_{j}$ are given by:

(5) $$a_{j}={\left[a_{j,1},\;a_{j,1},\cdots ,\;a_{j,n}\right]}^{T}$$

(6) $$\{b_{j}\}=\left\{\sqrt{\frac{n}{2(n-1)}}(a_{i}+a_{l}):i<l;i,l=1,2,\cdots ,n+1\right\}$$

where the vector elements of $a_{j}$ is defined as:

(7) $$a_{j,m}=\left\{ \begin{array}{l} \begin{array}{cc} -\sqrt{\frac{n+1}{n(n-m+2)(n-m+1)},} & m<j \end{array}\\ \begin{array}{cc} \sqrt{\frac{\left(n+1)(n-j+1\right)}{n(n-j+2)},} & \;m=j \end{array}\\ \begin{array}{cc} 0, & \;m>j \end{array} \end{array}\right.,\;1\leq m\leq n,\;1\leq j\leq n+1$$

#### 2.1.2. Radial Rule

The radial integral $R=\int_{0}^{\infty }S(r)r^{n-1}\exp(-r^{2})dr$ can be calculated by the following moment matching equation:

(8) $$\int_{0}^{\infty }S(r)r^{n-1}\exp(-r^{2})dr=\sum_{i=1}^{N_{r}}\omega_{r,i}S(r_{i})$$

where $S(r)=r^{l}$ is a monomial in *r*, with *l* an even integer. The left-hand side of Equation (8) is simplified as $\frac{1}{2}\Gamma \left(\frac{n+l}{2}\right)$ with Γ(n)=$\int_{0}^{\infty }x^{n-1}e^{-x}dx$. In order to achieve the fifth-degree algebraic precision, we make the radial integral *R* is exact for *l* = 0, 2, 4. For the fifth-degree radial rule $\left(N_{r}=2\right)$, we can obtain the moments’ equations as:

(9) $$\left\{ \begin{array}{l} \omega_{r,1}r_{1}^{0}+\omega_{r,2}r_{2}^{0}=\frac{1}{2}\Gamma \left(\frac{n}{2}\right)\\ \omega_{r,1}r_{1}^{2}+\omega_{r,2}r_{2}^{0}=\frac{1}{2}\Gamma \left(\frac{n+2}{2}\right)=\frac{n}{4}\Gamma \left(\frac{n}{2}\right)\\ \omega_{r,1}r_{1}^{4}+\omega_{r,2}r_{2}^{4}=\frac{1}{2}\Gamma \left(\frac{n+4}{2}\right)=\frac{1}{2}\left(\frac{n}{2}+1\right)\left(\frac{n}{2}\right)\Gamma \left(\frac{n}{2}\right) \end{array}\right.$$

By solving Equation (9), the points and weights for the third-degree radial rule are given by:

(10) $$\left\{ \begin{array}{l} r_{1}\;=0,\;r_{1}=\sqrt{n/2+1}\\ \omega_{r,1}=\frac{1}{n+2}\Gamma (n/2),\;\omega_{r,2}=\frac{n\Gamma (n/2)}{2(n+2)} \end{array}\right.$$

#### 2.1.3. Fifth-Degree Spherical Simplex-Radial Rule

By using Equations (3), (4) and (10), the fifth-degree spherical simplex-cubature rule can be formulated as:

(11) $$\begin{array}{l} \int_{{\mathbb{R}}^{n}}g(x)N(x;\;\hat{x},\;P_{x})dx=\frac{2}{n+2}g(\hat{x})+\\ \;\{\frac{(7-n)n^{2}}{2{\left(n+1\right)}^{2}{\left(n+2\right)}^{2}}\sum_{i=1}^{n+1}\left[g(\sqrt{(n+2)P_{x}}a_{i}+\hat{x})+g(-\sqrt{(n+2)P_{x}}a_{i}+\hat{x})\right]+\\ \;\frac{2{\left(n-1\right)}^{2}}{2{\left(n+1\right)}^{2}{\left(n+2\right)}^{2}}\sum_{i=1}^{n(n+1)/2}\left[g(\sqrt{(n+2)P_{x}}b_{i}+\hat{x})+g(-\sqrt{(n+2)P_{x}}b_{i}+\hat{x})\right]\} \end{array}$$

The steps of 5thSSRCKF algorithm for the nonlinear system can be found in [22,25].

## 3. IMM5thSSRCKF Algorithm

**Figure 1:**
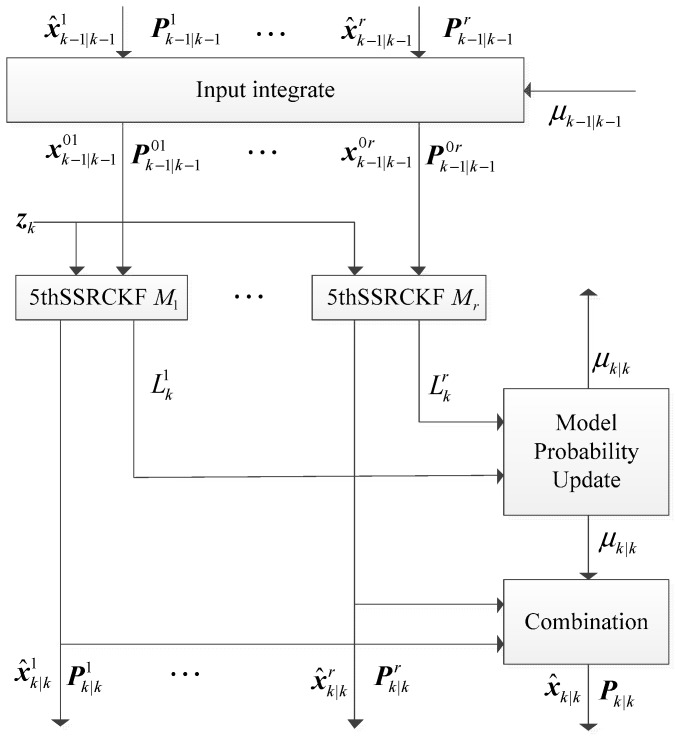
Structure of interacting multiple model fifth-degree spherical simplex-radial cubature Kalman filter (IMM5thSSRCKF).

The IMM algorithm obtains the output state estimate as a weighted sum of the estimates from a number of filters. In the application of IMM algorithm, the filtering precision depends on the selected sub-filter. Considering that 5thSSRCKF has high estimation precision, 5thSSRCKF is selected as sub-filter in the filtering part of the IMM framework. Therefore, the proposed IMM5thSSRCKF algorithm is the combination of IMM algorithm and 5thSSRCKF algorithm. In the IMM5thSSRCKF algorithm, the state estimation at time *k* is computed under each possible current model using *r* filters, with each filter using a different combination of the previous model-conditioned estimates. The structure diagram of IMM5thSSRCKF is shown in Figure 1.

In the IMM5thSSRCKF algorithm, the 5thSSRCKF employs the fifth-degree spherical simplex-radial cubature rule to generate the cubature points, which can further estimate the mean and covariance of the system state. The IMM5thSSRCKF algorithm includes four fundamental steps: model interaction, model conditional filtering, model probability updating, and output integration. The detailed steps of the IMM5thSSRCKF algorithm are provided as follows.

### **Step 1.** Model Interaction

The initial condition for each model j can be obtained from the state estimate ${\hat{x}}_{k-1|k-1}^{j}$ and covariance $P_{k-1|k-1}^{j}$ at time k−1. The mixed initial state of model j at time k−1 ${\hat{x}}_{k-1|k-1}^{0j}$ and its corresponding covariance $P_{k-1|k-1}^{0j}$ are computed according to:

(12) $$\begin{array}{l} {\hat{x}}_{k-1|k-1}^{0j}=\sum_{i=0}^{r}\mu_{k-1|k-1}^{i|j}{\hat{x}}_{k-1|k-1}^{i}\\ P_{k-1|k-1}^{0j}=\sum_{i=0}^{r}\mu_{k-1|k-1}^{i|j}\{P_{k-1|k-1}^{i}+\left[{\hat{x}}_{k-1|k-1}^{i}-{\hat{x}}_{k-1|k-1}^{0j}\right]{\left[{\hat{x}}_{k-1|k-1}^{i}-{\hat{x}}_{k-1|k-1}^{0j}\right]}^{T}\} \end{array}$$

where *r* denotes the number of interacting models, and ${\hat{x}}_{k-1|k-1}^{i}$ and $P_{k-1|k-1}^{i}$ are the prior state estimate and corresponding state error covariance of model i in the previous step, respectively.

The mixing probability $\mu_{k-1|k-1}^{i|j}$ at time k−1 can be given by:

(13) $$\begin{array}{l} \mu_{k-1}^{i|j}=\frac{p_{ij}\mu_{k-1}^{i}}{C_{j}},\\ C_{j}=\sum_{i=1}^{r}p_{ij}\mu_{k-1}^{i} \end{array}$$

where i,j=1,⋯,r, $\mu_{k-1}^{i}$ represents the model probability of the mode *i* at time *k*−1, and $p_{ij}$ is the probability of a transition from model *i* to model j.

### **Step 2.** Model Conditional Filtering

Using the initial mixing state $x_{k-1|k-1}^{0j}$ and the covariance $P_{k-1|k-1}^{0j}$ of the interacting step as the input of each filter at time k−1. Then, the new state ${\hat{x}}_{k|k}^{j}$ of model *j* and covariance $P_{k|k}^{j}$ of model j can be updated by Equations (14)–(20).

#### A. Time Update

The evaluation of cubature points in the mechanism of state one-step prediction and the propagated cubature points in the mechanism of state one-step prediction can be obtained by the following equations:

(14) $$\begin{array}{l} X_{i,k|k-1}^{j}=S_{k-1|k-1}^{0j}\xi_{i}^{j}+x_{k-1|k-1}^{0j}\\ X_{i,k|k-1}^{*j}=f(X_{i,k|k-1}^{j}) \end{array}$$

where $S_{k-1|k-1}^{0j}$ is the square root factor of $P_{k-1|k-1}^{0j}$, and $P_{k-1|k-1}^{0j}$ is the estimated error covariance of model j at time k−1. $\left\{\xi_{i}^{j}\right\}$ is the matrix with a set of vector, and the corresponding weight matrix is $\left\{\omega_{i}^{j}\right\}$. The fifth-degree simplex cubature points and the corresponding weights are as follows:

(15) $$\xi_{i}^{j}=\left\{ \begin{array}{ll} {\left[0,0,\cdots ,0\right]}^{T} & i=1,\\ \sqrt{n+2}a_{i-1} & i=2,\cdots ,n+2,\\ -\sqrt{n+2}a_{i-n-2} & i=n+3,\cdots ,2n+3,\\ \sqrt{n+2}b_{i-2n-3} & i=2n+4,\cdots ,(n^{2}+5n+6)/2,\\ -\sqrt{n+2}b_{i-(n^{2}+5n+6)/2} & i=(n^{2}+5n+8)/2,\cdots ,n^{2}+3n+3. \end{array}\right.$$

(16) $$\omega_{i}^{j}=\left\{ \begin{array}{ll} \frac{2}{n+2} & i=1,\\ \frac{(7-n)n^{2}}{2{\left(n+1\right)}^{2}{\left(n+2\right)}^{2}} & i=2,\cdots ,2n+3,\\ \frac{2{\left(n-1\right)}^{2}}{n{\left(n+1\right)}^{2}{\left(n+2\right)}^{2}} & i=2n+4,\cdots ,n^{2}+3n+3. \end{array}\right.$$

where *n* represents the dimension of state vector; the point sets of $a_{i}$ and $b_{i}$ are given by (5) and (6).

The predicted state $x_{k|k-1}^{j}$ and predicted error covariance $P_{k|k-1}^{j}$ can be computed using the cubature transformation as:

(17) $$\begin{array}{l} x_{k|k-1}^{j}=\sum_{i=1}^{L}\omega_{i}X_{i,k|k-1}^{*j}\\ P_{k|k-1}^{j}=\sum_{i=1}^{L}\omega_{i}(X_{i,k|k-1}^{*j}-x_{k|k-1}^{j}){\left(X_{i,k|k-1}^{*j}-x_{k|k-1}^{j}\right)}^{T}+Q_{k-1} \end{array}$$

where the number of points *L* is $n^{2}+3n+3$, and $Q_{k-1}$ denotes the system noise covariance matrix.

#### B. Measurement Update

The cubature points used for the measurement update and the propagated cubature points are derived as:

(18) $$\begin{array}{l} \chi_{i,k|k-1}^{j}=S_{k|k-1}^{j}\xi_{i}^{j}+x_{k|k-1}^{j}\\ Z_{i,k|k-1}^{j}=h(\chi_{i,k|k-1}^{j}) \end{array}$$

where $S_{k|k-1}^{j}$ can be obtained by factorizing the predicted error covariance $P_{k|k-1}^{j}$.

The prediction value of the measurement vector $z_{k|k-1}^{j}$, the innovation covariance matrix $P_{zz,k|k-1}^{j}$, and the cross covariance matrix $P_{xz,k|k-1}^{j}$ are given as follows:

(19) $$\begin{array}{l} z_{k|k-1}^{j}=\sum_{i=1}^{L}\omega_{i}Z_{i,k|k-1}^{j}\\ P_{zz,k|k-1}^{j}=\sum_{i=1}^{L}\omega_{i}(Z_{i,k|k-1}^{j}-z_{k|k-1}^{j}){\left(Z_{i,k|k-1}^{j}-z_{k|k-1}^{j}\right)}^{T}+R\\ P_{xz,k|k-1}^{j}=\sum_{i=1}^{L}\omega_{i}(\chi_{i,k|k-1}^{j}-x_{k|k-1}^{j}){\left(Z_{i,k|k-1}^{j}-z_{k|k-1}^{j}\right)}^{T} \end{array}$$

Finally, the estimated state ${\hat{x}}_{k|k}^{j}$ of model j and the estimated error covariance $P_{k|k}^{j}$ of model j can be derived as follows:

(20) $$\begin{array}{l} K_{k}^{j}=P_{xz,k|k-1}^{j}\cdot \mathrm{inv}(P_{zz,k|k-1}^{j})\\ {\hat{x}}_{k|k}^{j}=x_{k|k-1}^{j}+K_{k}^{j}(z_{k}-z_{k|k-1}^{j})\\ P_{k|k}^{j}=P_{k|k-1}^{j}-K_{k}^{j}P_{zz,k|k-1}^{j}{\left(K_{k}^{j}\right)}^{T} \end{array}$$

### **Step 3.** Updating the Mode Probability at Time k

#### **A.** **Computing the likelihood function at time *k***

With the use of the latest measurement $z_{k}$, the likelihood function value of model j at time k is given by:

(21) $$L_{k}^{j}=N(z_{k};z_{k|k-1}^{j},v_{k}^{j})={\left|2\pi S_{k}^{\left(j\right)}\right|}^{-n_{z}/2}\exp\left\{-\frac{1}{2}{\left[z_{k}-z_{k|k-1}^{j}\right]}^{T}{\left(S_{k}^{\left(j\right)}\right)}^{-1}[z_{k}-z_{k|k-1}^{j}]\right\}$$

where $v_{k}^{j}=z_{k}-z_{k|k-1}^{j}$ denotes the filter residual and $S_{k}^{\left(j\right)}$ denotes the innovation covariance and $n_{z}$ denotes the dimension of measurement vector.

#### **B.** **Updating the mode probability at time *k***

The mode probability $\mu_{k|k}^{j}$ at time k is computed by the following equation:

(22) $$\mu_{k|k}^{j}=\frac{L_{k}^{j}C_{j}}{C},\;C=\sum_{i=1}^{r}L_{k}^{j}C_{i}$$

### **Step 4.** Output Integration

Finally, the state estimate ${\hat{x}}_{k|k}$ and corresponding covariance $P_{k|k}$ are obtained by the model-conditional estimates and covariances of different models:

(23) $${\hat{x}}_{k|k}=\sum_{j=1}^{r}\mu_{k|k}^{j}\;{\hat{x}}_{k|k}^{j}$$

(24) $$P_{k|k}=\sum_{j=1}^{r}\mu_{k|k}^{j}\;\left\{P_{k|k}^{j}+\left[{\hat{x}}_{k|k}^{j}-{\hat{x}}_{k|k}\right]{\left[{\hat{x}}_{k|k}^{j}-{\hat{x}}_{k|k}\right]}^{T}\right\}$$

## 4. Simulation and Results

To validate the performance of the proposed algorithm, a highly maneuvering target example has been considered. The proposed algorithm will be compared with the IMMCKF, IMMUKF, and IMM5thCKF algorithm.

### 4.1. Tracking Model and Measurement Model

Let the state vector at time *k* be $x_{k}={\left[x_{k},{\dot{x}}_{k},y_{k},{\dot{y}}_{k}\right]}^{T}$, which includes the position (m) and velocity component (m/s) in the x-axis and y-axis. For tracking of the maneuvering target, three models are employed: the constant velocity (CV) model, left constant turn (LCT) model and right constant turn (RCT) model. For constant velocity model, the equation of state is described as:

(25) $$x_{k}=F_{\mathrm{CV}}x_{k-1}+w_{\mathrm{CV}},$$

(26) $$F_{\mathrm{CV}}=\left[\begin{array}{cccc} 1 & T & 0 & 0\\ 0 & 1 & 0 & 0\\ 0 & 0 & 1 & T\\ 0 & 0 & 0 & 1 \end{array}\right]$$

where $w_{\mathrm{CV}}$ is the white Gaussian process noise with zero mean and nonsingular covariance $Q_{\mathrm{CV}}$.

(27) $$Q_{\mathrm{CV}}=\left[\begin{array}{cccc} T^{3}/3 & T^{2}/2 & 0 & 0\\ T^{2}/2 & T & 0 & 0\\ 0 & 0 & T^{3}/3 & T^{2}/2\\ 0 & 0 & T^{2}/2 & T \end{array}\right]q_{\mathrm{CV}}$$

where the scalar parameter $q_{\mathrm{CV}}$ is the spectral density and set to 1. The constant turn (CT) model is defined as:

(28) $$x_{k}=F_{\mathrm{CT}}x_{k-1}+w_{\mathrm{CT}},$$

(29) $$F_{\mathrm{CT}}=\left[\begin{array}{cccc} 1 & \frac{\sin\omega T}{\omega } & 0 & -\left(\frac{1-\cos\omega T}{\omega }\right)\\ 0 & \cos\omega T & 0 & -\sin\omega T\\ 0 & \frac{1-\cos\omega T}{\omega } & 1 & \frac{\sin\omega T}{\omega }\\ 0 & \sin\omega T & 0 & \cos\omega T \end{array}\right]$$

where $w_{\mathrm{CT}}$ is the white Gaussian process noise with zero mean and nonsingular covariance $Q_{\mathrm{CT}}$.

(30) $$Q_{\mathrm{CT}}=\left[\begin{array}{cccc} T^{3}/3 & T^{2}/2 & 0 & 0\\ T^{2}/2 & T & 0 & 0\\ 0 & 0 & T^{3}/3 & T^{2}/2\\ 0 & 0 & T^{2}/2 & T \end{array}\right]q_{\mathrm{CT}}$$

where the scalar parameter $q_{\mathrm{CT}}$ is set to 1, *T* is the sampling interval, *w* stands for the turn rate which is supposed to be known, the right turn rate is defined as −3°, and the left turn rate is defined as 3°.

In the experiment, the radar is located at the origin of the plan and equipped to measure range and bearing. Then, the measurement equation can be written as:

(31) $$z_{k}=\left(\begin{array}{c} r_{k}\\ \theta_{k} \end{array}\right)=\left(\begin{array}{l} \sqrt{x_{k}^{2}+y_{k}^{2}}\\ \tan^{-1}(y_{k}/x_{k}) \end{array}\right)+v_{k}$$

where $r_{k}$ is the range value at time *k*, $\theta_{k}$ is the bearing value at time *k*, tan^−1^(·) is the inverse tangent function, and $v_{k}$ is the white Gaussian measurement noise with zero mean and covariance $R_{k}=diag([\sigma_{r}^{2},\sigma_{\theta }^{2}])$. $\sigma_{r}$ and $\sigma_{\theta }$ denote the standard deviation of range measurement noise and bearing angle measurement noise, respectively.

### 4.2. Simulation of the IMM5thSSRCKF

The simulation scene is designed as follows. The sampling interval is *T* = 1 s and repeats 100 times. The target moves in different state for five periods. The initial position is (15,000 m, 1000 m) and the target starts at 1 s with the velocity (−180 m/s, 200 m/s). From 1 s to 20 s it has motion at constant velocity; from 21 s to 70 s it turns right with ω=−3°; from 71 s to 120 s it has motion at a constant velocity; from 121 s to 170 s it maneuvers and turns left with ω=3°; and from 171 s to 200 s it has motion at constant velocity.

The initial estimates ${\hat{x}}_{0}$ are generated from the Gaussian distribution $N({\hat{x}}_{0};x_{0},P_{0})$ in which the true initial is $x_{0}$ = [15,000, −180, 100, 200]*^T^*. The standard deviation of range measurement noise $\sigma_{r}$ is 40 m and the standard deviation of bearing angle measurement noise $\sigma_{\theta }$ is 7 mrad. The initial model probability is μ = [0.8 0.1 0.1] and the transition probability is given as:

(32) $$p_{ij}=\left[\begin{array}{ccc} 0.95 & 0.025 & 0.025\\ 0.025 & 0.95 & 0.025\\ 0.025 & 0.025 & 0.95 \end{array}\right]$$

The root mean square error (RMSE) of the target position at time *k* and the accumulative RMSE (ARMSE) of estimated position at all times are defined in Equations (33) and (34):

(33) $${\mathrm{RMSE}}_{\mathrm{pos}}(k)=\sqrt{\frac{1}{M}\sum_{m=1}^{M}\left({\left(x_{k}-{\hat{x}}_{m,k}\right)}^{2}+{\left(y_{k}-{\hat{y}}_{m,k}\right)}^{2}\right)}$$

(34) $${\mathrm{ARMSE}}_{\mathrm{pos}}=\sqrt{\frac{1}{N}\sum_{k=1}^{N}\left({\mathrm{RMSE}}_{\mathrm{pos}}^{2}(k)\right)}$$

where M is the number of Monte Carlo runs, *N* is the total number of sampling points, $\left(x_{k},y_{k}\right)$ is the actual value of the target position at time k and $\left({\hat{x}}_{m,k},{\hat{y}}_{m,k}\right)$ is the estimated position at time k in *m*th Monte-Carlo. The RMSE and the accumulative RMSE in the velocity and acceleration can be defined in the same way. The performance comparison of the four algorithms are tested 200 times in Monte Carlo simulations.

**Figure 2:**
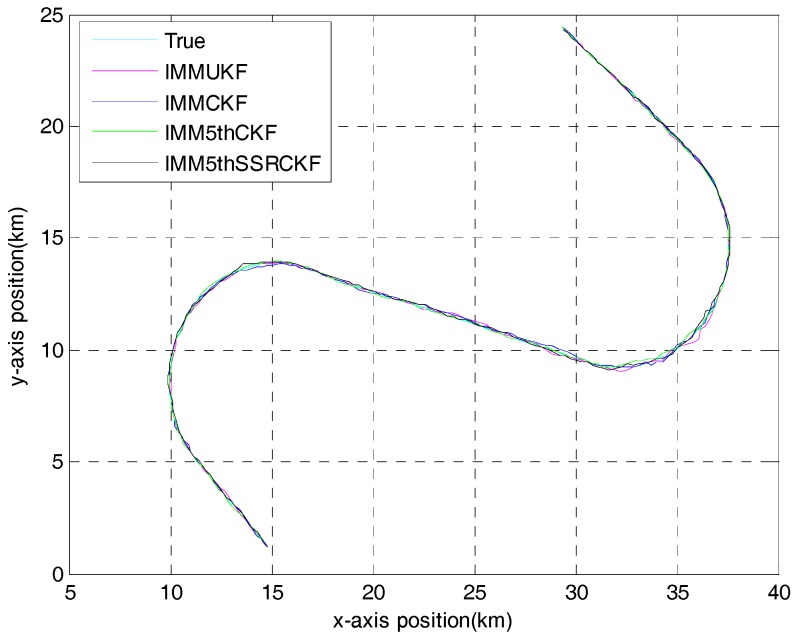
Trajectory of the maneuvering target. IMMUKF: interacting multiple model unscented Kalman filter; IMMCKF: interacting multiple model cubature Kalman filter; IMM5thCKF: interacting multiple model fifth-degree cubature Kalman filter; IMM5thSSRCKF: interacting multiple .model fifth-degree spherical simplex-radial cubature Kalman filter

Figure 2 gives the target trajectory and the state estimation generated from a single run of IMMUKF, IMMCKF, IMM5thCKF and IMM5thSSRCKF. As seen from Figure 2, these four algorithms can track the trajectory of the target.

**Figure 3:**
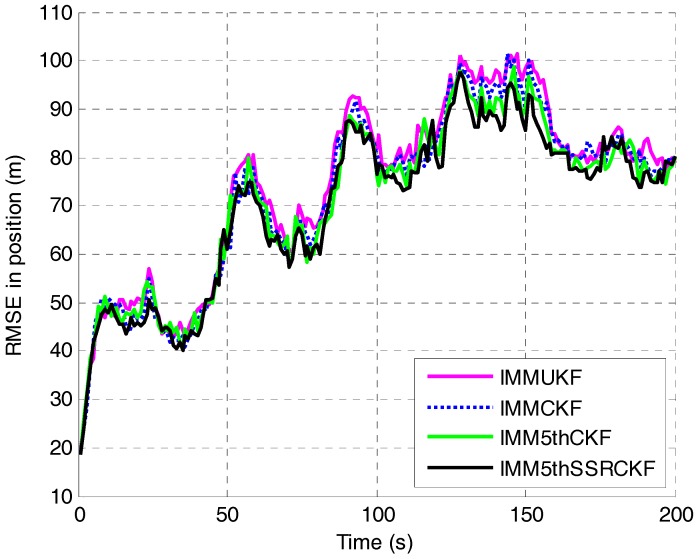
RMSE in position versus time step.

**Figure 4:**
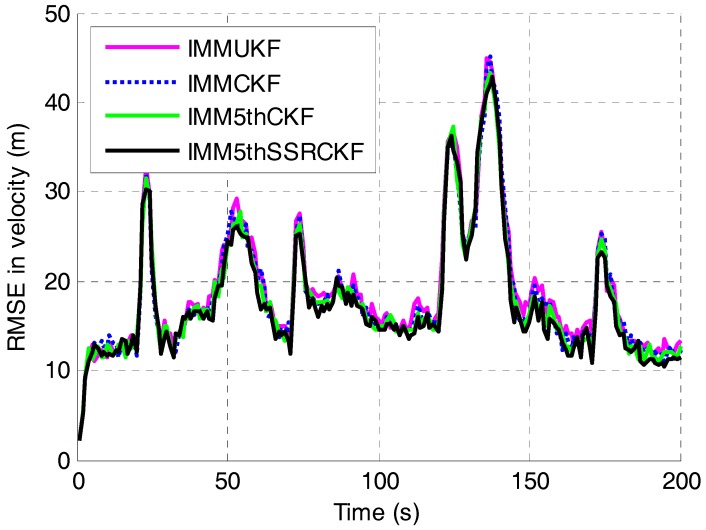
RMSE in velocity versus time step.

The RMSEs in position and velocity of the four algorithms are shown in Figure 3 and Figure 4, respectively. It can be seen that the proposed IMM5thSSRCKF performs better than the IMMUKF, IMMCKF and IMM5thCKF algorithms when the target moves with CV. The tracking error of target position of the three IMM algorithms would be almost the same when the target moves at constant velocity. The estimation effectiveness of the IMM5thSSRCKF estimator for tracking a maneuvering target outperform greatly than the other two IMM estimators.

To further evaluate the performance of the four algorithms, the ARMSEs of position and velocity of each algorithm are listed in Table 1. It can be seen from the Table 1 that IMM5thSSRCKF does better in tracking precision than IMMUKF, IMMCKF and IMM5thCKF, while all of them exhibit no error divergence.

**Figure 5:**
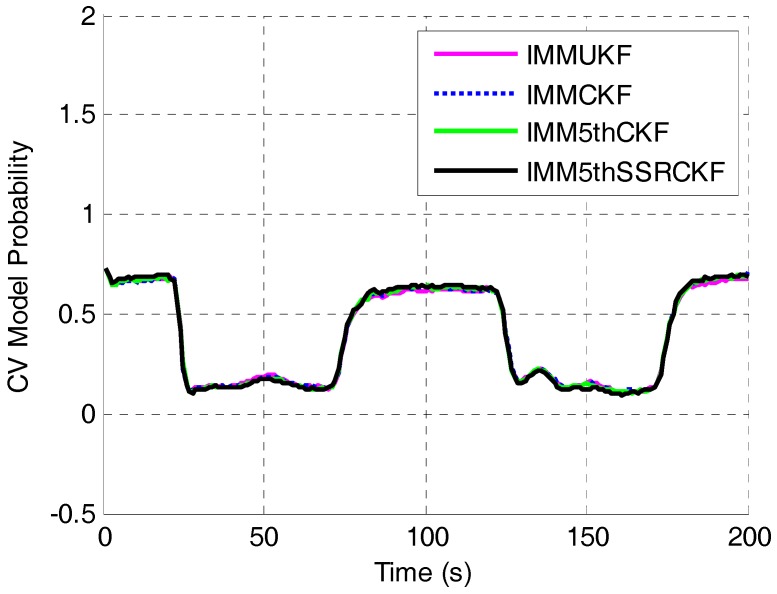
Constant velocity (CV) mode probability versus time step.

The comparisons of CV mode probability of IMMUKF, IMMCKF, IMM5thCKF and IMM5thSSRCKF are shown in Figure 5. The mode transitions occur at *t* = 21 s, *t* = 71 s, *t* = 121 s and *t* = 171 s, respectively. This figure shows that the IMMUKF, IMMCKF, IMM5thCKF and IMM5thSSRCKF can capture the kinematics of maneuvering when the motion state changes. It can be seen that the mode probabilities of the IMMUKF algorithm are not good at detecting mode transitions. The proposed algorithm and IMM5thCKF algorithm are equally faster at detecting model changes compared with the IMMUKF algorithm and the IMMCKF algorithm.

All the algorithms are implemented on the Intel Core^TM^ i5-4430 3.0GHZ CPU with 4.00 G RAM. Table 2 shows the number of points and computational time of IMMUKF, IMMCKF, IMM5thCKF and IMM5thSSRCKF for each run. The points of IMMCKF as well as IMMUKF differ only by one point. As shown in Table 2, the computational time of the algorithms is approximately proportional to the number of points. It is obvious that the IMM5thSSRCKF algorithm has a slightly lower computational cost than the IMM5thCKF due to the different cubature rule. Although the computation complexity of IMM5thSSRCKF algorithm is larger than IMMUKF and IMMCKF, it can be remedied by more high-speed computer technology.

## 5. Conclusions

Maneuvering target tracking is the research hot spot in the target tracking field; this paper has presented a new maneuvering target tracking algorithm named IMM5thSSRCKF. The 5thSSRCKF algorithm is an efficient method to deal with the problem of nonlinear system estimation. The proposed algorithm introduces the 5thSSRCKF algorithm into the IMM framework, which can dispose of all the models simultaneously through Markov Chain. The performance of the proposed method is evaluated by simulations and compared with IMMUKF, IMMCKF and IMM5thCKF. Simulation results illustrate that the IMM5thSSRCKF algorithm has higher tracking accuracy and a quicker sensitivity response than IMMUKF, IMMCKF and IMM5thCKF algorithms.

## Figures and Tables

**Table 1:** Comparisons of accumulative RMSE (ARMSE) among the four algorithms.

Filters	Position ARMSE/m	Velocity ARMSE/(m/s)
IMMUKF	74.3	23.4
IMMCKF	72.4	22.5
IMM5thCKF	68.1	20.9
IMM5thSSRCKF	66.2	19.3

**Table 2:** Number of points and computational time of different algorithms.

Filters	Number of Points (*n =* 4)	Computational Time (s)
IMMUKF	9	0.289
IMMCKF	8	0.279
IMM5thCKF	33	0.604
IMM5thSSRCKF	31	0.581
